# Clinicopathological Studies on the Impact of Grape Seed Extract and L-Carnitine as Cardioprotective Agents Against Doxorubicin-Induced Toxicity in Rats

**DOI:** 10.3390/life14121656

**Published:** 2024-12-13

**Authors:** Tahany Saleh Aldayel, Omnia E. Kilany, Heba Nageh Gad El-Hak, Heba M. A. Abdelrazek, Osama Abdallah, Donia E. Omar

**Affiliations:** 1 Department of Health Sciences, Clinical Nutrition, College of Health and Rehabilitation Sciences, Princess Nourah bint Abdulrahman University, Riyadh 11671, Saudi Arabia; TSALdayel@pnu.edu.sa; 2 Department of Clinical Pathology, Faculty of Veterinary Medicine, Suez Canal University, Ismailia 41522, Egypt; omniakilany@vet.suez.edu.eg (O.E.K.); dr_oabdallah@hotmail.com (O.A.); 3 Depatrment of Zoology, Faculty of Sciences, Suez Canal University, Ismailia 41522, Egypt; heba_ahmed@science.suez.edu.eg; 4 Department of Physiology, Faculty of Veterinary Medicine, Suez Canal University, Ismailia 41522, Egypt; heba_abdelrazek@vet.suez.edu.eg

**Keywords:** cardiotoxicity, doxorubicin, grape seed extract, L-Carnitine

## Abstract

Doxorubicin (DOX) cancer therapy induces serious cardiotoxicity as a side effect. This study aimed to investigate the cardioprotective effects of grape seed extract (GSE) and L-Carnitine (L-CA) against DOX-induced cardiac toxicity in male rats. Six groups of male albino rats were used: G1 (control); G2 (GSE), given grape seed extract (100 mg/kg b.wt.) orally for 35 days; G3 (L-CA) (150 mg/kg b.wt.); Group 4 (DOX-induced cardiotoxicity), given DOX (10 mg/kg b.wt., i.p.) on the 28th day of the experiment; G5 (GSE + DOX), given GSE and DOX as previously mentioned; and G6 (L-CA + DOX), given L-CA and DOX as previously mentioned. Electrocardiographic evaluation, lipid profile, lipid peroxidation and antioxidants, serum cardiac markers, and inflammatory markers were estimated. Histopathological evaluation of cardiac tissue was also examined. Key findings showed that DOX induced ECG abnormalities lipid peroxidation, reduced antioxidants, and elevated cardiac and inflammatory markers. GSE and L-CA significantly ameliorated ECG abnormalities, reduced lipid peroxidation, improved antioxidant enzymes and serum cardiac markers, and reduced inflammation. These findings suggest that GSE and L-CA exhibit substantial cardioprotective effects in DOX-induced cardiotoxicity via their antioxidant and anti-inflammatory potentials.

## 1. Introduction

Cancer is one of the main causes of death all over the world [1]. Despite the undeniable successes of chemotherapy, two critical problems remain unresolved: resistance to anticancer drugs and their associated toxicity [2]. One of the most significant drawbacks of anticancer therapy is its cardiovascular side effects, which can sometimes require reducing the drug dosage to ineffective levels or even discontinuing the treatment, posing severe health risks [3]. Doxorubicin (DOX), a potent cytotoxic antibiotic, falls under the anthracycline class and is developed from the *Streptomyces peucetius* var. *caesius* mutant variant as a secondary metabolite [4]. DOX is highly effective against a wide range of cancers, including malignancies of hematological origin like non-Hodgkin’s lymphomas, Hodgkin’s disease, and pediatric leukemia, as well as solid tumors such as those in the bladder, breast, and lung [5]. Nevertheless, DOX’s use is restricted due to its dramatic adverse effects, which include vomiting, hair loss, nausea, hematological suppression, and a unique cardiotoxicity [6].

The mechanism behind DOX-induced heart failure involves a significant increase in free radicals and a decline in myocardial endogenous antioxidant activities [7]. This imbalance renders cardiac tissues particularly vulnerable to oxidative damage owing to their diminished antioxidant enzyme levels. Furthermore, DOX has a strong affinity for the phospholipids in the heart cells’ mitochondrial membranes, resulting in its deposition in cardiac tissues. This accumulation triggers damage to intracellular components and the myocardium, as well as their membranes [8].

Mitigating heart damage caused by DOX could be achieved through treatments aimed at reducing oxidative stress [9]. Hence, there has been an increasing interest in using natural antioxidants as a preventive measure against heart issues [10]. Numerous polyphenols possessing substances with cardioprotective and antioxidant properties have recently been recognized. They can prevent DOX-induced injury to myocardial function and structure in rats by lowering the production of free radicals, increasing mitochondrial function, and reducing apoptosis [7].

Grapes, known for their rich polyphenol content, including proanthocyanidins (PAs), anthocyanins, and resveratrol, offer a diverse array of biological benefits, including anti-inflammatory, antioxidant, antidiabetic, antitumor, and cardioprotective effects [11]. Preclinical experiments have demonstrated that PAs can protect against the harmful effects of DOX on cardiac tissue. This protective effect is linked to a reduction in blood markers for cardiac damage and oxidative stress in the myocardium of experimental animals [12].

Carnitine (3-hydroxy-4-N-trimethylaminobutyrate), a small water-soluble molecule similar to a vitamin, is found in nearly all mammalian species [13]. Its primary role is to transport long-chain fatty acids in active form from the cellular cytosol to the matrix of the mitochondria, where β-oxidation happens [14]. L-Carnitine (L-CA) has several antioxidant mechanisms, including the capacity to quench free radicals directly; chelate catalytic metals-promoters of reactive oxygen species (ROS), like Cu and Fe; keep the integrity of the mitochondria; hinder ROS generation; and prohibit ROS-generating enzymes like NADPH oxidases and xanthine oxidase with further generation of antioxidant enzymes [15]. Grapes are one of the most widely cultivated fruits worldwide, owing to their diverse applications, including fresh consumption and the production of wine, grape juice, jam, and raisins. However, the massive manufacture of grape products produces large masses of byproducts, such as grape pomace and grape seeds, which can pose environmental problems and represent a major waste of valuable resources. There has been a growing interest in using these byproducts due to their rich composition of polyphenols. These compounds are renowned for their diverse beneficial properties [11], including anti-inflammatory, antioxidant, antidiabetic, antitumor, and cardioprotective effects [11]. On the other hand, L-CA is widely used as a nutritional supplement and is available in several forms, such as glycine-propionyl L-CA and L-CA L-tartrate; it has gained popularity for its role in enhancing athletic performance and improving muscle recovery. Its recovery-enhancing effects are attributed to its ability to boost the activity of antioxidant enzymes [16], which can effectively mitigate exercise-induced muscle damage [17].

The present study was designed to investigate the potential cardioprotective influences of GSE and L-CA against DOX-induced cardiac damage in male rats. This aim was achieved through the detection of electrocardiographic changes, lipid profile, lipid peroxidation and antioxidants, serum cardiac markers, and inflammatory markers, which were estimated. Also, the histopathological evaluation of cardiac tissue was examined, as well as the cardiac fibrous tissue installation.

## 2. Materials and Methods

### 2.1. Animals 

Sixty male albino rats (300–400 g) were housed in the Laboratory Animal House of the Faculty of Veterinary Medicine at Suez Canal University, Egypt. The rats were allocated randomly into six groups, with ten rats per group (five rats per cage). To allow acclimation, they were kept under standard conditions, including room temperature (24 ± 2 °C), a natural daylight cycle, and free access to a basal diet for one week. Throughout the 35-day experimental duration, the rats were maintained at a room temperature of 24 ± 2 °C and a 55 ± 5% relative humidity. The experimental procedures were authorized by the institutional review board of the Faculty of Veterinary Medicine, Suez Canal University (protocol No. 2022013). 

### 2.2. Plant Material, L-Carnitine, and Doxorubicin

Grape seed extract was obtained from Shaanxi Jintai Biological Engineering Co., Ltd. (Xi’an, China) (CAS No.: 84929-27-1). L-Carnitine was sourced from MARTINEZ NIETO (Murcia, Spain)in the form of 2000 mg liquid L-Carnitine tartrate. Doxorubicin hydrochloride (Adriadox^®^) was purchased from RMPL Pharma LLP (Mumbai, Maharashtra, India) in a 50 mg vial. 

### 2.3. Study Design

The experimental rats were allocated randomly into six groups (ten in each) as follows:

Group 1 (control) was considered as the normal control group. Five rats received oral CMC, and the other five rats received distal water via gavage for 35 days. All of them were injected with IP saline on the 28th day.

The Group 2 (GSE) rats were given 100 mg/kg b.wt. of 1% *w*/*v* CMC via gavage daily for 35 days. The dosage was determined according to Nassiri-Asl and Hosseinzadeh [18]. The Group 3 (L-CA) rats received 150 mg/kg b.wt. of 33.3% *v*/*v* via gavage twice weekly for 35 days, with the dosage according to Tousson, Hafez [19].

In Group 4 (DOX), the cardiotoxicity was provoked by an IP injection of 10 mg/kg b.wt. DOX on the 28th experimental day. The dosage used to induce cardiotoxicity followed the protocol of Adıyaman, Adıyaman [9].

The Group 5 (GSE + DOX) rats received 100 mg/kg b.wt. of 1% *w*/*v* CMC via gavage daily for 35 days, and on the 28th experimental day, the rats were injected IP with 10 mg/kg b.wt. DOX. 

The Group 6 (L-CA + DOX) rats received 150 mg/kg b.wt. of 33.3% *v*/*v* via gavage twice per week for 35 days, and on the 28th experimental day, the rats were injected IP with 10 mg/kg b.wt. DOX. 

### 2.4. Phytochemical Analysis of Grape Seed Extract

Phytochemical analysis was performed using an Agilent 1260 series. The separation was carried out using an Eclipse C18 column (4.6 mm × 250 mm i.d., 5 μm). The mobile phase consisted of water (A) and 0.05% trifluoroacetic acid in acetonitrile (B) at a flow rate of 0.9 mL/min. The mobile phase was programmed consecutively in a linear gradient as follows: 0 min (82% A); 0–5 min (80% A); 5–8 min (60% A); 8–12 min (60% A); 12–15 min (82% A); 15–16 min (82% A); and 16–20 (82% A). The multi-wavelength detector was monitored at 280 nm. The injection volume was 5 μL for each of the sample solutions. The column temperature was maintained at 40 °C. The quantification method was carried out using the following standards: catechin, gallic acid, caffeic acid, methyl gallate, pyro catechol, syringic acid, ellagic acid, chlorogenic acid, rutin, coumaric acid, vanillin, naringenin, ferulic acid, quercetin, daidzein, apigenin, cinnamic acid, kaempferol, and hesperetin.

### 2.5. Electrocardiography (ECG)

ECG recordings were taken for all the experimental groups. The experimental animals were fully anaesthetized through tetrahydrofuran (THF) inhalation. Electrodes of ECG were attached subcutaneously to the paws of the rats where they were positioned on their backs. The electrodes were connected to a Kaden Yasen™ ECG-903 device (Zhuhai City, Guangdong, China). The recorded ECG parameters included heart rate (HR), QT and QRS complex intervals, and ST segment amplitude. The later parameters were obtained as four readings/rats in all the groups. All the rats (5 rats/group) were subjected to ECG analyses on the 29th day and 35th day.

### 2.6. Blood and Sample Collection

Rats were sacrificed twice: on day 29 (24 h post–cardiotoxicity induction) and day 35 (experiment completed). Blood was drawn from the retro-orbital sinus under mild THF anesthesia after an overnight fast. The blood samples were processed for biochemical, antioxidant, and cytokines analyses. The rats were subsequently euthanized with an overdose of THF. The hearts were divided for histological analysis and stored at −80 °C for oxidant and antioxidant assays. Cardiac tissue homogenates were prepared using the Lowry protein assay described by Lu, Yiao [20].

### 2.7. Determination of Lipid Profile

Serum triglycerides (TG), total cholesterol (TC), and high-density lipoprotein cholesterol (HDL-C) were determined using Clinchem kits (Hungary) according to the manufacturer’s protocols [21,22,23], respectively. Low-density lipoprotein cholesterol (LDL-C) (1)and very low-density lipoprotein cholesterol (VLDL-C) (2) were calculated via the equations described by Obasi and Ogugua [24]. (1) Serum LDL-C (mg/dL) = TC − HDL-C − TG/5. (2) Serum VLDL-C (mg/dL) = TG/5.

### 2.8. Cardiac Lipid Peroxidation and Antioxidants

Catalase (CAT), reduced glutathione (GSH), malondialdehyde (MDA), and total nitric oxide (TNO) were measured by kits from Cell Biolabs, Inc. (San Diego, CA, USA), following the manufacturer’s protocols [25,26,27], respectively. The total oxidative capacity (TOC) and total antioxidative capacity (TAC) were evaluated using kits from Labor Diagnostika Nord GmbH & Co. KG (Nordhorn, Germany), adhering to the protocols provided by the manufacturer [28]. The procedures of the kits were carried out according to [29,30]. In addition, LOOH was assayed using the Cayman Chemical kits’ protocol [25]. 

### 2.9. Determination of Serum Cardiac Marker 

Creatine Kinase (CK-MB), lactate dehydrogenase (LDH), and aspartate aminotransferase (AST) activities in serum were measured using Clinchem kits (Budapest, Hungary) according to the manufacturer’s protocols [31,32,33]. Serum cardiac troponin I (cTnI) and NT-proBNP were measured using ELISA kits from Kamiya Biomedical Company (Seattle, WA, USA) and Cusabio Technology LLC (Wuhan, China) in accordance with the manufacturer’s guidelines [34,35].

### 2.10. Inflammatory Markers 

The ELISA kits for measuring serum IL-1β, TNF-α (Kamiya Biomedical Company, Seattle, WA, USA), MPO, and NF-Kß were obtained from (Cusabio Technology LL-C, Wuhan, China). The assay procedures were followed as the kit manufacturer’s protocol. 

### 2.11. Histopathological Examination of the Heart Tissue

Samples of the heart tissues were put in 10% neutral buffered formalin for fixation, subjected to dehydration via passage of graded alcohol concentration, subjected to xylene clearing, and immersed in paraffin. Sections 5 microns in thickness were subjected to staining via Masson’s trichrome (on day 35) and Hematoxylin and Eosin (H&E) (on day 29 and day 35) for light microscopic examination according to Bancroft and Cook [36]. Four random fields per slide were imaged to determine the percentage area of collagen fibers in the Masson’s trichrome slides. A total of 3 slides/rats were examined. 

### 2.12. Statistical Analysis 

The data from the study were tested using the Shapiro–Wilk test, which revealed that all the data were normal except the ECG parameters and that the percentage area of collagen fibers was not found to be parametric. The Kruskal–Wallis H test was performed to determine the differences in the later parameters between the groups. Additionally, SPSS 29 employed a violin plot of the ECG data. The rest of the study data were analyzed via SPSS version 25. The results were expressed as mean ± SE. One-way ANOVA followed by Tukey’s test was used for analysis, with *p* < 0.05 considered statistically significant.

## 3. Results

### 3.1. Phytochemical Analysis of Grape Seed Extract 

**Figure 1:**
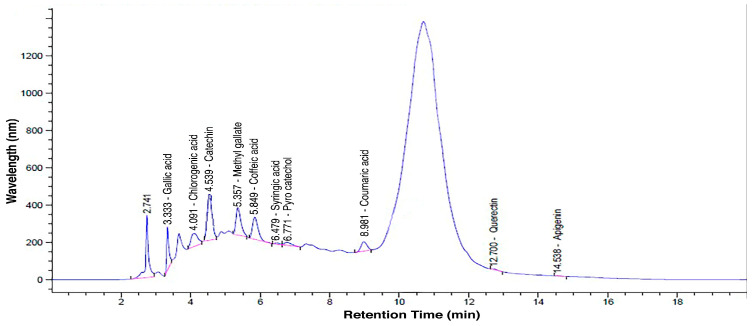
Phytochemical analysis of grape seed extract.

The phytochemical screening of the standard laboratory procedures revealed the presence of phenolic compounds (chlorogenic acid, catechin, gallic acid, methyl gallate, pyrocatechol, caffeic acid, syringic acid, coumaric acid, quercetin, and apigenin) (Table 1, Figure 1). The highest concentration among the active compounds was observed in catechin, with a content of 11,385.07 µg/g, while the lowest concentration was in apigenin, with a content of 12.91 µg/g.

### 3.2. ECG

**Figure 2:**
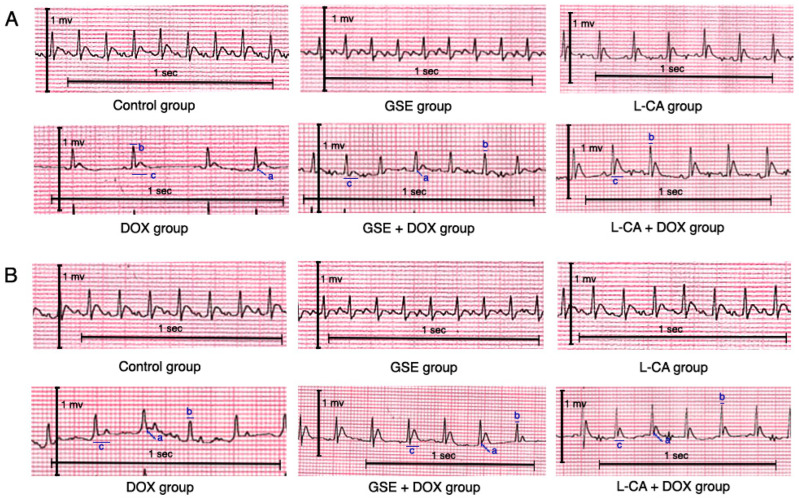
(**A**) Representative ECG on day 29 of the experiment. (**B**) Representative ECG on day 35 of the experiment. Group GSE: grape seed extract, Group L-CA: L-Carnitine; Group DOX: Doxorubicin; Group GSE + DOX: Grape seed extract with Doxorubicin, Group L-CA+ DOX: L-Carnitine with Doxorubicin. Labels a, b, and c denote the following ECG parameters: (a) ST-segment elevation, (b) QRS interval and (c) QT interval.

**Figure 3:**
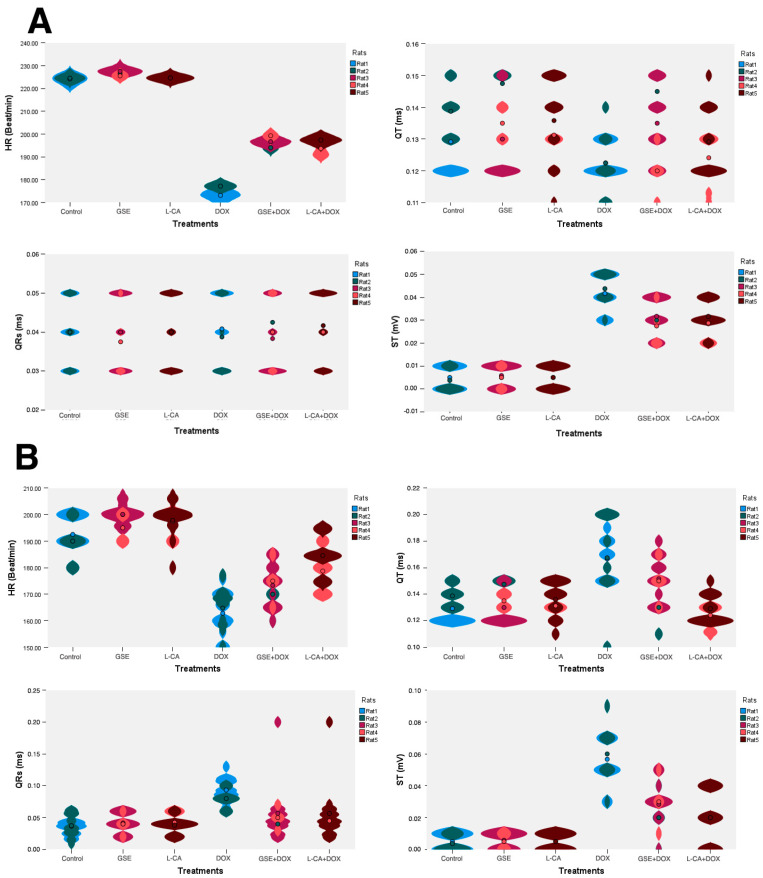
The impact of doxorubicin, grape seed extract, and L-carnitine on heart rate (HR), QRS complex, QT interval, and ST segment amplitude. (**A**) Representation of the changes in ECG on day 29 of the experiment (*n* = 5). Data for each rat were obtained from 4 readings/rat. (**B**) Representation of the changes in ECG on day 35 of the experiment (*n* = 5). Data for each rat were obtained from 4 readings/rat.

As seen in (Figure 2 and Figure 3), there were no statistical differences among the control, GSE, and L-CA groups in both the 29th and 35th days of the experiment. The DOX group exhibited a marked decrease in HR and a statistical elevation in the ST segment. On the 29th day, there were non-significant changes in the QT and QRS complex intervals, but by the 35th day, these intervals were significantly elongated in comparison to the control group. The groups pretreated with GSE and L-CA demonstrated a significant increase in HR and a significant decrement in ST segment amplitude in comparison to the DOX group on both days. However, there were no significant changes in the QT and QRS complex intervals on the 29th day. By the 35th day, the data revealed no significant change in the QRS complex, while a statistically significant shortening of the QT interval was noted in comparison to the DOX rats.

### 3.3. Lipid Profile

As mentioned in Table 2, the present results demonstrated non-significant variations in TG, TC, HDL-C, LDL-C, and VLDL-C levels in the groups that received GSE and L-CA in comparison to the control group on the 29th and 35th days of the experimental duration. In contrast, DOX injection resulted in a significant increase in TG, TC, LDL-C, and VLDL-C and a significant decline in HDL-C in comparison to the control rats on the 29th and 35th days. Pretreatment with GSE and L-CA led to significant improvements in these parameters compared to the DOX group, with a notable decrease in TG, TC, LDL-C, and VLDL-C and a statistical promotion in HDL-C. Moreover, the L-CA pretreated group showed a significant reduction in TC and LDL-C on the 29th and 35th days, along with improvements in TG and VLDL-C levels on day 35, compared to the rats pretreated with GSE.

### 3.4. Cardiac Lipid Peroxidation and Antioxidants

As declared in Table 3, the present study revealed a non-significant alteration in lipid peroxidation and antioxidant levels in the experimental groups orally administered with GSE and L-CA when compared to the control group on both day 29 and day 35 of the experiment. In contrast, the group intoxicated with DOX displayed a significant decline in GSH, CAT, and TAC, along with a significant increase in TOC, MDA, TNO, and LOOH on both days compared to the control group.

Moreover, the groups pretreated with GSE and L-CA demonstrated significant enhancements in GSH, CAT, and TAC, as well as a statistical reduction in TOC, MDA, and TNO on both days compared to the DOX group. Specifically, the group pretreated with L-CA showed an improvement in CAT, MDA, TNO, and LOOH on the 29th and 35th days, along with a significant increase in TAC on day 35 of the experiment.

### 3.5. Cardiac Injury Markers

As demonstrated in Table 4, the activity of CK-MB, LDH, AST, cTnI, and NT-ProBNP showed non-significant variations in the groups treated with GSE and L-CA as compared with the control group on both the 29th and 35th days of the experimental duration. In contrast, DOX administration provoked a notable increase in serum cardiac markers compared to the control group on both days. However, significant diminution in these markers was consistently detected in the groups pretreated with GSE and L-CA compared to the DOX group, suggesting a protective effect against DOX-induced cardiac injury. Furthermore, L-CA pretreatment exhibited significant reductions in cardiac injury markers compared to the GSE-pretreated group on both days, indicating a potentially superior protective effect of L-CA.

### 3.6. Cardiac Inflammatory Markers

As demonstrated in Table 5, the levels of IL-1β, TNF-α, MPO, and NF-Kß showed non-significant variations in the groups treated with GSE and L-CA compared to the control group on both day 29 and day 35 of the experiment. However, the DOX-injected group exhibited a statistical increase in the levels of IL-1β, TNF-α, MPO, and NF-Kß in comparison to the control group at both time intervals. Additionally, the groups pretreated with GSE and L-CA displayed a significant decrease in these markers compared to the DOX group on the 29th and 35th days. Notably, the group pretreated with L-CA demonstrated a more significant decrease in IL-1β, TNF-α, MPO, and NF-Kß levels compared to the group pretreated with GSE on the 29th and 35th days.

### 3.7. Histopathology on Cardiac Tissue Sections

**Figure 4:**
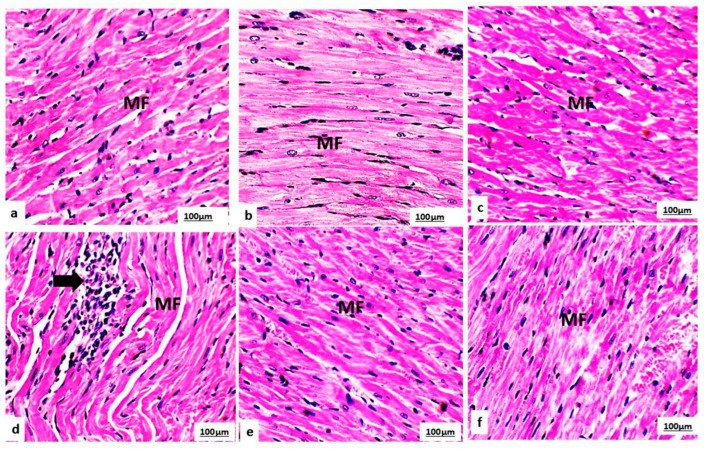
Photomicrographs of cardiac myocytes from the control, GSE, and L-CA groups (**a**–**c**) on day 29 of the experiment displayed normal cardiac myofibrillar structure (MF) with striations, branched appearance, central nucleus, and continuity with adjacent myofibrils. In contrast, the DOX group (**d**) revealed several abnormalities, including nuclear pyknosis and leukocyte infiltration (indicated by arrows). The DOX + GSE and DOX + L-CA groups (**e**,**f**) showed a restoration to normal cardiac myofibrillar structure (C) with clear striations. (H&E, 400×).

**Figure 5:**
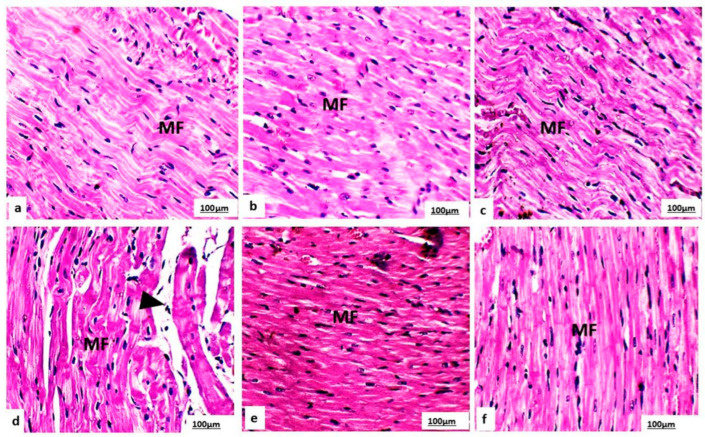
Photomicrographs of cardiac myocytes from the control, GSE, and L-CA groups (**a**–**c**) on day 35 of the experiment showed normal cardiac myofibrillar structure (MF) with striations, a branched appearance, a central nucleus, and continuity with adjacent myofibrils. In contrast, the DOX group (**d**) exhibited signs of myocardial infarction, including focal myolysis, non-visible nuclei, and darker, irregular necrotic contraction bands (indicated by arrowheads). The DOX + GSE and DOX + L-CA groups (**e**,**f**) displayed a return to normal cardiac myofibrillar structure (C) with clear striations. (H&E, 400×).

**Figure 6:**
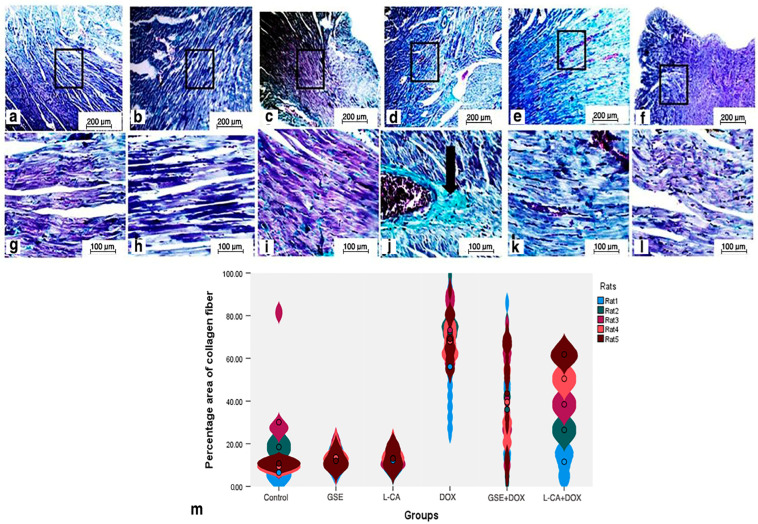
Photomicrographs of Masson-trichrome-stained cardiac myocytes of low and high magnification from the control (**a**,**g**), GSE (**b**,**h**), and L-CA groups (**c**,**i**) on day 35 of the experiment depicted normal cardiac myofibrillar structure with striations, a branched appearance, a central nucleus, and continuity with adjacent myofibrils. In contrast, the DOX group (**d**,**j**) exhibited interstitial fibrosis, with an increase in the percentage area of collagen fiber (arrow). However, the GSE + DOX (**e**,**k**) and L-CA + DOX groups (**f**,**l**) displayed a restoration of normal cardiac myofibrillar structure with clear striations, accompanied by a decrease in the percentage area of collagen fiber. (100× & 400×). The mean percentage area of collagen fiber (**m**) was quantified across (*n* = 5) rats/group. Each set of rat data was obtained from 12 measurements. Group GSE: grape seed extract; Group L-CA: L-Carnitine; Group DOX: Doxorubicin; Group GSE + DOX: Grape seed extract with Doxorubicin; Group L-CA + DOX: L-Carnitine with Doxorubicin.

Light microscopy of the rat cardiac sample tissue sections on the control, L-CA, and GSE groups on day 29 (Figure 4a–c) and day 35 (Figure 5a–c) of the experiment revealed normal cardiomyocytes and typical connective tissue on day 35 (Figure 6a–c). The cardiac wall consists of striated, branched cardiomyocytes containing intercalated disks and typically mononucleated nuclei.

Histopathological examination of the cardiac sample tissue sections on the DOX group on day 29 (Figure 4d) and day 35 (Figure 5d) revealed several abnormalities, including degeneration of the myofibrillar structure, myocardial atrophy, nuclear pyknosis, focal hemorrhage, edema, leukocyte infiltration, and interstitial fibrosis. There was an increased percentage area of collagen fibers on day 35, as evidenced by Masson trichrome staining (Figure 6d).

In contrast, the groups pretreated with L-CA or GSE on both day 29 (Figure 4e,f), and day 35 (Figure 5e,f) showed a marked reduction in the abnormalities observed in the DOX group. The heart tissue from these pretreated groups appeared to have normal myocardial muscle bundles with no interstitial fibrosis and a decreased percentage area of collagen fibers, as indicated by the Masson trichrome staining (Figure 6e,f).

## 4. Discussion

Doxorubicin is a highly effective chemotherapeutic agent extensively cast off in cancer treatment. However, its clinical use is significantly limited by its cardiotoxic side effects. DOX-induced cardiotoxicity is primarily linked to increased inflammation, oxidative stress, and apoptosis in cardiac tissue [37]. In the present study, we evaluated the possible alleviation of GSE and L-CA in DOX-induced cardiotoxicity.

Cardiotoxicity was confirmed by significant changes across multiple parameters, including ECG findings, serum biomarkers indicative of oxidative stress, cardiac injury, and histopathological analysis. Our results declared that DOX statistically reduced HR and caused alterations in ECG waves, including prolonged QRS duration and QT interval, as well as elevated ST segment amplitude. The reduction in HR was consistent with Prathumsap, Ongnok [38], who attributed the bradycardia to sinoatrial node dysfunction and disruptions in cardiac conduction caused by cell death and oxidative stress. However, Ammar, Said [39] found a contradictory result and reported an increase in HR shortly after DOX treatment, likely as a reflex to acute cardiomyopathy from a higher DOX dose (15 mg/kg, i.p.). The observed prolongation of the QRS duration and QT interval was in line with Sheibani, Nezamoleslami [40] and Sobhy, Ismail [41], suggesting that DOX-induced significant heart damage led to these abnormalities. Additionally, the elevated ST segment, a key marker of cardiomyocyte membrane damage, further confirmed the extent of cardiac injury. This was supported by the increased levels of cardiac enzymes, oxidative stress, and histopathological findings, all of which indicate underlying membrane damage and highlight the severity of DOX-induced cardiotoxicity.

The present study demonstrated that both GSE and L-CA exhibit cardioprotective effects against DOX-induced ECG abnormalities and help protect cell membranes from damage. Ammar, Said [39] suggested that GSE stabilizes cell membranes by limiting the interaction of lipid peroxyl radicals with nearby membrane polyunsaturated fatty acids, thus impeding the propagation phase of lipid peroxidation. Similarly, Mustafa, Hegazy [42] reported that L-CA’s antioxidant properties reduce oxidative stress and promote membrane stabilization in cardiomyocytes. The active ingredients of GSE such as catechin may be incriminated in such effects, and ECG improvement as previously reported by Schön, Allegrini [43]. The protective effects of GSE were further supported by the decline in cardiac enzyme levels, improvements in oxidative status, and the restoration of both the functional and structural integrity of the myocardium. 

In the DOX group, our study found significantly high levels of VLDL-C, TG, LDL-C, and TC with a significant decline in HDL-C. Similar findings were reported by Al-Sowayan [44] and Sobhy, Ismail [41]. According to Boghdady [45], DOX-induced hyperlipidemia is likely linked to nephrotic syndrome, which results from DOX-induced glomerulonephritis. The hyperlipidemia observed in our study is an important marker of DOX-induced metabolic disruption and correlates with cardiotoxicity.

The administration of GSE and L-CA, however, resulted in significant improvements in the lipid profile, indicating their ability to mitigate the adverse effects of DOX on the lipid metabolism. Previous studies declared the anti-hyperlipidemic influence of GSE phenolic constituents such as gallic acid [46], chlorogenic acid, caffeic acid [47], syringic acid [48], and coumaric acid [49]. Phenolic compounds are known to prevent TG absorption from the gut and stimulate lipoprotein lipase activity [50]. Moreover, flavonoids such as quercetin [51], catechin [52], apigenin [53], pyro catechol [54], and methyl gallate [55] possess ani-hyperlipidemic effects. L-CA, on the parallel side, was found to reduce DOX’s harmful effects on lipid levels, as supported by Meky, Haggag [56]. L-CA could perform as a burner for body fat that encourages the lipids’ metabolism and declines the blood LDL-C [57]. Additionally, Martín, Giráldez [58] suggested that L-CA decreases cholesterol levels by reducing acetyl coenzyme A, a precursor for cholesterol synthesis, as free carnitine binds with acetyl coenzyme A to form acetylcarnitine. 

DOX-induced oxidative stress emerged as a key factor contributing to cardiac injury, as proved by raised levels of MDA and depletion of antioxidant enzymes. These findings align with Aziz, Abd El Fattah [59]. Birari, Wagh [60] attributed this effect to the quinone and hydroquinone groups within the DOX structure facilitating the formation of semiquinone radical intermediates. These intermediates intensify oxidative stress, leading to the depletion of the body’s antioxidant reserves. Sheibani, Nezamoleslami [40] further supported this mechanism, highlighting that DOX cardiotoxicity results from an imbalance between ROS and antioxidant enzyme levels. The excessive ROS generated by DOX not only disrupts this balance but also causes significant cellular damage, leading to increased lipid peroxidation and MDA formation. Ogonowski, Mikusic [61] added that DOX cardiotoxicity can be amplified under metabolic perturbations or in other health ailments that comprise cardiovascular risk factors.

Our study revealed that both GSE and L-CA significantly improved the cardiac antioxidant defense system. This protective effect is consistent with their antioxidant properties. The greatest reduction was observed with L-CA treatment, followed by GSE, which suggests their potential role in mitigating DOX-induced cardiotoxicity by enhancing the heart’s antioxidant defenses. 

The protective effect of GSE is attributed to the rich composition of bioactive compounds, which includes a variety of phenolic ingredients, such as chlorogenic acid, caffeic acid, syringic acid, and gallic acid as well as flavonoids such as quercetin, catechin, and apigenin, as was demonstrated in our phytochemical analysis. These findings reflect the contribution of specific polyphenols and flavonoids in the antioxidant activity of GSE. GSE exerts its antioxidant effect by stabilizing cell membranes and facilitating electron transfer in oxidation-reduction reactions, thus neutralizing reactive species. GSE’s ability to inhibit enzymes such as cyclooxygenase, lipoxygenase, and phospholipase A2, as well as its metal-chelating properties, further reduce oxidative damage [62]. The high catechin content identified in our phytochemical analysis exerts protective effects on vascular endothelial cells by inhibiting NADPH oxidase, an enzyme responsible for generating ROS [63]. Previous reports confirmed the antioxidative potential of the detected GSE’s ingredients on cardiomyocytes; Xu, Yang [64] displayed that apigenin could reduce cardiomyocytes’ oxidative stress parameters via modulating the SIRT1 pathway. Badavi, Sadeghi [65] demonstrated that gallic acid improved the antioxidant capacity of cardiomyocytes and enhanced the cell membrane integrity of rats’ myocytes after ischemic perfusion. Chlorogenic acid was found to reduce the oxidative stress induced by myocardial injury in myocardial-infarction-induced Sprague Dawley male rats [66]. Another study, by Wang, Kaur [67], revealed that caffeic acid abridged the atherosclerogenic-diet-induced oxidative damage on cardiomyocytes via MDA reduction and free radicals’ chelation in rats. Synergic acid can reduce oxidative stress cardiomyopathy in diabetic rats via its antioxidant potential [67]. Coumaric acid possesses antioxidant and anti-inflammatory influences on cardiac tissue that prevent its damage [68]. Methyl gallate was found to reverse DOX-induced MDA elevations and GSH depletion via its antioxidant potential in female rats [69]. Flavonoid constituents of GSE such as quercetin were previously found to diminish DOX-induced myocardial oxidative stress and injury in male rats [70]. Catechin is another GSE flavonoid that was proven to enhance antioxidant criteria in cardiomyocytes of SOD-knocked out mice [71]. 

In our research, the antioxidant properties of L-CA emerged as a significant protective factor against oxidative stress. This aligns with the verdicts of Sharma, Schmidt [72], who found that L-CA is a potent antioxidant and free radicals scavenger, protecting cells from oxidative stress and mitigating mitochondrial toxicity. This protective effect is achieved not only by neutralizing free radicals but also by promoting β-oxidation, which reduces the harmful impact of free fatty acids on cells. Furthermore, Meky, Haggag [56] found that L-CA reduces hydroxyl radical production, which is a key driver of oxidative damage, particularly during the Fenton reaction. This is achieved through the chelation of iron, a critical cofactor in hydroxyl radical formation.

Moreover, L-CA can inhibit NADPH oxidase, a source of superoxide anion in cardiomyocytes that protects cardiomyocytes against DOX-induced oxidative damage [73]. These mechanisms likely contribute to the overall cardioprotective effects of L-CA observed in our study. Sayed-Ahmed, Shaarawy [74] added that L-CA protects the heart against myocardial injury induced via DOX without changing DOX antitumor potentials by suppression of palmitate oxidation [74].

In our study, the DOX group had a statistical upsurge in cardiac injury biomarkers such as CK-MB, LDH, AST, cTnI, and NT-proBNP, all of which are released from damaged cardiomyocytes and serve as sensitive indicators of DOX-induced cardiotoxicity. This increase is likely attributable to the cardiac fiber lesions observed in the histopathological analysis. These outcomes were in agreement with Sobhy, Ismail [41] and Aziz, Abd El Fattah [59]. Bin Jardan, Ansari [8] demonstrated that DOX triggers the accumulation of free radicals within cardiac tissue, leading to oxidative damage to the myocardial walls. This damage compromises the integrity of myocardial cell membranes, causing the release of cardiac enzymes into the bloodstream. However, our findings of cTnI contradict those of Botelho, Lempek [75], whose results were negative for all the tested animals. Samples from the animals that got a single dosage after 7 days of DOX injury may have had reduced detection levels and thus were declared negative. 

Serum biochemical analysis provided valuable insights into the cardioprotective potential of GSE and L-CA. Our findings demonstrated that both GSE and L-CA statistically abridged the elevated serum echelons of cardiac enzymes induced by DOX. Nazima, Manoharan [76] explained that GSE achieves this by stabilizing cellular membranes and donating hydrogen from its phenolic compounds to neutralize free radicals. Similarly, Mustafa, Hegazy [42] attributed that to the lessening of oxidative stress biomarkers and the restoration of cardiomyocyte membrane integrity. Several studies confirmed that the active constituents of GSE abridged serum cardiac injury biomarkers and inflammatory markers. Gallic acid induced significant reduction in LDH and CK-MB as a result of antioxidant potential against DOX administration in male rats [10]. Chlorogenic acid induced significant amelioration of CK-MB, LDH, and cTnI in an induced myocardial ischemic injury model of 57 BL/6 mice [77]. They added that chlorogenic acid possesses an anti-inflammatory effect against myocardial injury via suppressing NLRP3 inflammasome and Neat1. The study by Zare, Rakhshan [78] showed that apigenin significantly ameliorated LDH, CK-MB, cTnI, and AST in DOX-induced-cardiac-injury male rats due to antioxidant, anti-inflammatory, and antiapoptotic potentials. Oktar, Aydın [79] showed that administration of caffeic acid significantly reduced AST, LDH, MPO, and CK in the myocardial- injury rat model. Synergic acid [80] and coumaric acid [81] induced antioxidant and anti-lipid peroxidation influence in myocardial-infarction-induced adult male rats that resulted in CK-MB, LDH, and AST reduction. Sharma, Parikh [82] demonstrated that quercetin ameliorated the DOX-induced cardiac toxicity, which statistically changed the cardiac biomarkers and improved myocardial histology. Ahmed, Satyam [69] demonstrated that methyl gallate reduced CK-MB, LDH, AST, MDA, and GSH levels in DOX-induced cardiotoxicity female Wistar rats with amelioration of DOX-induced ECG changes. Moreover, Aziz, Abd El Fattah [59] claimed the L-CA antioxidant potential for the reduction in cardiomyocytes damage and leakage of their enzymes into blood, whereas oxidative stress plays a central role in cardiomyocyte damage. 

The significant upregulation of inflammatory markers, including IL-1β, TNF-α, MPO, and NF-Kß, in the DOX-treated group suggests an inflammatory response to oxidative damage. This aligns with the results of Aziz, Abd El Fattah [59]. Sadek, Mahmoud [83] explained that oxidative stress caused by DOX-induced cardiac injury results in endothelial cell damage, leading to leukocytes infiltration and the release of pro-inflammatory cytokines. 

GSE and L-CA demonstrated protective effects by significantly reducing the levels of these markers in sera as matched with the DOX-intoxicated group. This suggests that both GSE and L-CA possess anti-inflammatory properties, but L-CA exhibited a more pronounced effect. The study’s findings showed that, in comparison to the intoxicated group, pre-treatment with GSE and L-CA caused a substantial drop in the serum echelons of TNF-α, MPO, NF-Kß, and IL-1β. These findings align with Zhou, Li [11], who demonstrated that grape-derived compounds are highly effective in reducing inflammation by inhibiting pro-inflammatory cytokine release and regulating associated signaling pathways. Feringa, Laskey [84] also demonstrated that flavonoids in GSE inhibit the lipoxygenase pathways responsible for generating pro-inflammatory leukotrienes, and help balance cytokine patterns, thus offering anti-inflammatory effects. Regarding L-CA anti-inflammatory action, Fallah and Mahdavi [85] and Aziz, Abd El Fattah [59] suggested that L-CA’s anti-inflammatory effects are primarily mediated by its ability to reduce ROS production, which in turn inhibits pro-inflammatory signaling, particularly the NF-Kß-mediated pathway. 

The histopathological analysis provided visual confirmation of the biochemical and ECG findings, revealing significant structural damage in the DOX-treated hearts, including myofibrillar degeneration, myocardial atrophy, and infiltration of inflammatory cells. These results are in line with the oxidative stress-induced damage reported by Aziz, Abd El Fattah [59] and Sadek, Mahmoud [83]. Sheibani, Nezamoleslami [40] showed that there was a correlation between the increase in CK-MB in blood, a diagnostic marker with a high predictive value for myocardial damage, and substantial heart histopathology damage. The DOX-induced cardiomyocyte damage could be attributed to DOX triggering to catecholamine release, which promotes p53-release and macrophages-dependent mitochondrial apoptosis [86]. In addition, DOX accumulates in the mitochondria and extinguishes the mitochondrial electron chain, which provokes ROS production [7]. Moreover, DOX induces BAX production that leads to both apoptosis and necrosis of myocardial cells [87], therefore leaking their enzymes into circulation and causing histopathological damage. 

The observed increase in collagen fiber deposition and interstitial fibrosis further supports the role of oxidative stress in promoting fibroblast proliferation. This aligns with Mustafa, Hegazy [42], who reported that cardiac stress promotes the proliferation of myofibroblasts, which are α-SMA positive and play a crucial part in the progression of fibrosis, causing the structural changes noticed in DOX-induced cardiac injury.

Pretreatment with GSE and L-CA resulted in marked histological improvements, with a significant reduction in myofibrillar damage, edema, and inflammation. Al-Sowayan [44] suggested that GSE’s phenolic compounds, particularly catechins and proanthocyanidins, have been shown to enhance endogenous antioxidant defenses and prevent apoptosis, thus preserving cardiac tissue integrity. Moreover, GSE catechin and polyphenols were found to abridge DOX-induced cardiac damage by decreasing the cohort of ROS and the number of apoptotic cells, regulating the expression levels of the antiapoptotic protein Bcl-2 and the proapoptotic protein Bax α, impeding apoptotic signaling trails and averting DNA fragmentation [88]. Similarly, Tousson, Hafez [19] highlighted that L-CA and its derivatives are potent free radical scavengers, effectively reducing ROS production and protecting cells from oxidative stress. L-CA might provoke prostacyclin production, which is crucial for the anti-apoptotic effect that preserves cardiomyocytes against DOX damage [73]. This ability to preserve heart muscle structure and improve overall cardiac function further emphasizes the cardioprotective properties of both GSE and L-CA. 

While GSE and L-CA mitigated DOX-induced myocardial damage, the degree of protection appeared to differ between the two. The group pretreated with L-CA showed a more marked reduction in collagen fiber deposition and fibrosis compared to the group pretreated with GSE. According to Mustafa, Hegazy [42], this superior effect of L-CA may be attributed to its capability to hinder the transformation of fibroblasts into myofibroblasts, thereby limiting collagen deposition and the progression of cardiac fibrosis. 

These histopathological improvements align with the reductions in biochemical markers of oxidative stress and inflammation, suggesting that both GSE and L-CA effectively attenuate DOX-induced cardiotoxicity through a combination of antioxidant, anti-inflammatory, and membrane-stabilizing mechanisms.

## 5. Conclusions

Doxorubicin exerts cardiotoxic effects, as evidenced by ECG abnormalities, increasing oxidative stress, altered lipid profiles, elevated cardiac biomarkers, inflammation, and cardiac tissue damage. These impacts were effectively ameliorated via GSE and L-CA. Their cardioprotective effects were due to their antioxidant and anti-inflammatory possessions that improve cardiac biomarkers in sera.

## Figures and Tables

**Table 1:** Phytochemical analysis of grape seed extract.

Chemical Class	Compound	Wavelength (nm)	Retention Time (min)	Contents (µg/g)
Phenolic compounds	Gallic acid	280	3.333	1623.68
	Chlorogenic acid	280	4.091	2925.33
	Caffeic acid	280	5.843	1990.29
	Syringic acid	280	6.479	121.76
	Coumaric acid	280	8.981	342.14
	Pyro Catechol	280	6.771	719.14
Flavonoids	Quercetin	280	12.700	110.31
	Catechin	280	4.539	11,385.07
	Methyl Gallate	280	5.357	1692.11
	Apigenin	280	14.538	12.91

**Table 2:** The impact of doxorubicin, grape seed extract, and L-Carnitine on serum lipid profile on days 29 and 35 of the experiment.

Treatment		Triglycerides (mg/dL)	Total Cholesterol (mg/dL)	HDL-C (mg/dL)	LDL-C (mg/dL)	VLDL-C (mg/dL)
Control	Day 29	63.36 ^c^ ± 0.62	55.20 ^d^ ± 0.55	13.59 ^a^ ± 0.35	28.73 ^d^ ± 1.05	12.67 ^c^ ± 0.12
	Day 35	64.26 ^d^ ± 0.64	55.65 ^d^ ± 0.50	14.29 ^a^ ± 0.36	28.50 ^d^ ± 0.40	12.85 ^d^ ±0.13
GSE	Day 29	62.68 ^c^ ± 0.64	55.30 ^d^ ± 0.55	14.32 ^a^ ± 0.61	28.17 ^d^ ± 0.09	12.54 ^c^ ± 0.13
	Day 35	63.27 ^d^ ±0.64	54.49 ^d^ ± 0.64	14.60 ^a^ ± 0.64	27.23 ^d^ ± 0.20	12.65 ^d^ ± 0.13
L-CA	Day 29	62.39 ^c^ ± 0.53	55.60 ^d^ ± 0.61	14.63 ^a^ ± 0.55	26.23 ^d^ ± 0.24	12.48 ^c^ ± 0.11
	Day 35	63.18 ^d^ ± 1.01	54.24 ^d^ ± 0.49	14.86 ^a^ ±0.55	26.75 ^d^ ± 0.90	12.63 ^d^ ± 0.09
DOX	Day 29	107.32 ^a^ ± 0.61	92.20 ^a^ ± 0.55	8.67 ^c^ ± 0.68	49.20 ^a^ ± 1.24	21.46 ^a^ ± 0.12
	Day 35	100.87 ^a^ ± 0.58	84.40 ^a^ ± 0.53	9.70 ^c^ ± 0.21	44.64 ^a^ ± 1.21	20.17 ^a^ ± 0.12
GSE + DOX	Day 29	94.44 ^b^ ± 0.67	86.52 ^b^ ± 0.64	9.37 ^c^ ± 0.26	46.17 ^b^ ± 1.34	18.89 ^b^ ± 0.13
	Day 35	85.77 ^b^ ± 0.77	77.75 ^b^ ± 0.52	10.23 ^bc^ ± 0.23	41.25 ^b^ ± 0.56	17.15 ^b^ ± 0.14
L-CA + DOX	Day 29	95.55 ^b^ ± 0.67	80.37 ^c^ ± 0.64	11.27 ^b^ ± 0.37	39.70 ^c^ ± 1.31	19.11 ^b^ ± 0.13
	Day 35	83.26 ^c^ ± 0.64	68.33 ^c^ ± 0.61	11.30 ^b^ ± 0.23	33.02 ^c^ ± 0.10	16.65 ^c^ ± 0.13

Superscripts a, b, c, d within the same row are considered significant at *p* < 0.05. Group GSE: grape seed extract, Group L-CA: L-Carnitine; Group DOX: Doxorubicin; Group GSE + DOX: Grape seed extract with Doxorubicin, Group L-CA + DOX: L-Carnitine with Doxorubicin.

**Table 3:** The impact of doxorubicin, grape seed extract, and L-Carnitine on lipid peroxidation and antioxidant enzymes in the heart tissue on days 29 and 35 of the experiment.

Treatment		GSH (µM/mg)	CAT (U/mg)	TAC (mmol/g)	TOC (mmol/g)	MDA (µM/mg)	TNO (µM/mg)	LOOH (nmol/mg)
Control	Day 29	13.44 ^a^ ± 0.47	50.59 ^a^ ± 0.30	1.77 ^a^ ± 0.15	0.39 ^c^ ± 0.03	11.12 ^d^ ± 0.47	36.24 ^d^ ± 0.18	1.12 ^c^ ± 0.06
	Day 35	13.76 ^a^ ± 0.64	50.4 ^a^ ± 0.55	1.80 ^a^ ± 0.06	0.37 ^d^ ± 0.01	10.51 ^d^ ± 0.29	35.63 ^d^ ± 0.32	1.15 ^d^ ± 0.09
GSE	Day 29	13.88 ^a^ ± 0.52	51.31 ^a^ ± 0.38	1.86 ^a^ ± 0.19	0.40 ^c^ ± 0.02	11.35 ^d^ ± 0.60	35.78 ^d^ ± 0.15	1.15 c ± 0.03
	Day 35	13.80 ^a^ ± 0.67	50.6 ^a^ ± 0.61	1.81 ^a^ ± 0.06	0.37 ^d^ ± 0.01	10.32 ^d^ ±0.20	35.63 ^d^ ±0.32	1.15 ^d^ ± 0.09
L-CA	Day 29	13.95 ^a^ ± 0.52	51.61 ^a^ ± 0.31	1.85 ^a^ ± 0.03	0.39 ^c^ ± 0.02	11.25 ^d^ ± 0.65	35.71 ^d^ ± 0.21	1.16 ^c^ ± 0.04
	Day 35	14.59 ^a^ ± 0.61	52.4 ^a^ ± 0.69	1.82 ^a^ ± 0.06	0.35 ^d^ ± 0.01	10.11 ^d^ ± 0.25	35.61 ^d^ ± 0.31	1.15 ^d^ ± 0.09
DOX	Day 29	7.05 ^c^ ± 0.33	26.08 ^d^ ± 0.25	0.72 ^c^ ± 0.01	0.72 ^a^ ± 0.02	33.89 ^a^ ± 0.06	69.75 ^a^ ± 0.14	3.16 ^a^ ± 0.02
	Day 35	9.11 ^c^ ± 0.15	32.5 ^d^ ± 0.62	1.03 ^d^ ± 0.01	0.64 ^a^ ± 0.01	25.61 ^a^ ± 0.31	52.56 ^a^ ± 0.30	2.59 ^a^ ± 0.15
GSE + DOX	Day 29	8.61 ^b^ ± 0.35	31.42 ^c^ ± 0.30	1.03 ^b^ ± 0.01	0.67 ^ab^ ±0.03	28.55 b ± 0.83	58.24 ^b^ ± 0.30	3.18 ^a^ ± 0.36
	Day 35	10.56 ^b^ ± 0.12	41.9 ^c^ ± 0.58	1.33 ^c^ ± 0.01	0.53 ^b^ ± 0.01	19.36 ^b^ ± 0.20	46.46 ^b^ ± 0.29	2.08 ^b^ ± 0.10
L-CA + DOX	Day 29	9.49 ^b^ ± 0.29	36.33 ^b^ ± 0.35	1.28 ^b^ ± 0.01	0.61 ^b^ ± 0.03	25.36 ^c^ ± 0.41	52.43 ^c^ ± 0.23	2.43 ^b^ ± 0.03
	Day 35	11.77 ^b^ ± 0.15	45.5 ^b^ ± 0.61	1.63 ^b^ ± 0.01	0.42 ^c^ ± 0.01	13.77 ^c^ ± 0.15	39.17 ^c^ ± 0.29	1.50 ^c^ ± 0.12

Superscripts a, b, c, d within the same row are considered significant at *p* < 0.05. Group GSE: grape seed extract, Group L-CA: L-Carnitine; Group DOX: Doxorubicin; Group GSE + DOX: Grape seed extract with Doxorubicin, Group L-CA + DOX: L-Carnitine with Doxorubicin.

**Table 4:** The impact of doxorubicin, grape seed extract, and L-Carnitine on serum cardiac injury markers on days 29 and 35 of the experiment.

Treatment		CK-MB (U/L)	LDH (U/L)	AST (U/L)	cTnI (ng/mL)	NT-ProBNP (pg/mL)
Control	Day 29	14.52 ^c^ ± 0.87	235.23 ^d^ ± 2.90	121.13 ^d^ ± 2.31	0.21 ^d^ ± 0.01	9.75 ^d^ ± 0.03
	Day 35	15.10 ^d^ ± 0.52	238.30 ^d^ ± 0.05	123.47 ^d^ ± 0.61	0.21 ^d^ ± 0.00	9.50 ^d^ ± 0.40
GSE	Day 29	12.60 ^c^ ± 0.86	233.20 ^d^ ± 1.74	121.60 ^d^ ± 1.78	0.21 ^d^ ± 0.01	9.74 ^d^ ± 0.03
	Day 35	14.62 ^d^ ± 0.61	236.50 ^d^ ± 0.55	122.43 ^d^ ± 0.58	0.21 ^d^ ± 0.01	9.52 ^d^ ± 0.48
L-CA	Day 29	14.13 ^c^ ± 0.60	232.77 ^d^ ± 4.68	120.90 ^d^ ± 1.50	0.21 ^d^ ± 0.00	9.78 ^d^ ± 0.03
	Day 35	14.64 ^d^ ± 0.55	235.97 ^d^ ± 1.59	122.63 ^d^ ± 1.26	0.21 ^d^ ± 0.00	9.51 ^d^ ± 0.36
DOX	Day 29	32.95 ^a^ ± 1.16	574.77 ^a^ ± 2.90	285.47 ^a^ ± 2.49	1.19 ^a^ ± 0.03	45.63 ^a^ ± 2.88
	Day 35	26.73 ^a^ ± 0.61	506.57 ^a^ ± 0.66	232.67 ^a^ ± 0.55	0.88 ^a^ ± 0.01	37.60 ^a^ ± 0.35
GSE + DOX	Day 29	26.19 ^b^ ± 0.74	510.60 ^b^ ± 5.80	235.57 ^b^ ± 2.94	0.93 ^b^ ± 0.02	39.15 ^b^ ± 1.89
	Day 35	22.61 ^b^ ± 0.64	439.23 ^b^ ± 0.66	183.23 ^b^ ± 0.47	0.65 ^b^ ± 0.01	26.71 ^b^ ± 0.60
L-CA + DOX	Day 29	23.74 ^b^ ± 0.38	479.70 ^c^ ± 2.90	220.40 ^c^ ± 2.91	0.71 ^c^ ± 0.02	32.32 ^c^ ± 2.52
	Day 35	18.52 ^c^ ± 0.58	361.33 ^c^ ± 0.55	151.73 ^c^ ± 0.66	0.36 ^c^ ± 0.01	14.42 ^c^ ± 0.44

Superscripts a, b, c, d within the same row are considered significant at *p* < 0.05. Group GSE: grape seed extract, Group L-CA: L-Carnitine; Group DOX: Doxorubicin; Group GSE + DOX: Grape seed extract with Doxorubicin, Group L-CA + DOX: L-Carnitine with Doxorubicin.

**Table 5:** The impact of doxorubicin, grape seed extract, and L-Carnitine on cardiac inflammatory markers on days 29 and 35 of the experiment.

Treatment		IL-1β (pg/mg)	TNF-α (pg/mg)	MPO (ng/mg)	NF-Kß (pg/mg)
Control	Day 29	35.26 ^d^ ± 0.06	61.75 ^d^ ± 0.39	1.19 ^d^ ± 0.03	6.25 ^d^ ± 0.03
	Day 35	35.31 ^d^ ± 0.32	61.04 ^d^ ± 1.04	1.22 ^d^ ± 0.01	6.30 ^d^ ± 0.17
GSE	Day 29	35.34 ^d^ ± 0.24	62.12 ^d^ ± 0.58	1.19 ^d^ ± 0.02	6.22 ^d^ ± 0.03
	Day 35	34.60 ^d^ ± 0.32	60.54 ^d^ ± 0.29	1.21 ^d^ ± 0.00	6.25 ^d^ ± 0.14
L-CA	Day 29	34.84 ^d^ ± 0.23	61.49 ^d^ ± 0.29	1.19 ^d^ ± 0.02	6.19 ^d^ ± 0.03
	Day 35	34.51 ^d^ ± 0.31	60.07 ^d^ ± 0.23	1.20 ^d^ ± 0.01	6.16 ^d^ ± 0.09
DOX	Day 29	119.47 ^a^ ± 0.29	194.54 ^a^ ± 0.29	3.85 ^a^ ± 0.03	17.76 ^a^ ± 0.13
	Day 35	89.45 ^a^ ± 0.30	150.91 ^a^ ± 0.52	2.80 ^a^ ± 0.15	14.25 ^a^ ± 0.43
GSE + DOX	Day 29	91.81 ^b^ ± 0.23	163.53 ^b^ ± 0.29	3.25 ^b^ ± 0.03	15.19 ^b^ ± 0.04
	Day 35	73.36 ^b^ ± 0.29	117.40 ^b^ ± 0.35	2.27 ^b^ ± 0.18	12.16 ^b^ ± 0.60
L-CA + DOX	Day 29	76.12 ^c^ ± 0.07	134.22 ^c^ ± 0.12	2.65 ^c^ ± 0.03	13.75 ^c^ ± 0.03
	Day 35	53.55 ^c^ ± 0.29	78.41 ^c^ ± 0.42	1.81 ^c^ ± 0.14	8.53 ^c^ ± 0.46

Superscripts a, b, c, d within the same row are considered significant at *p* < 0.05. Group GSE: grape seed extract, Group L-CA: L-Carnitine; Group DOX: Doxorubicin; Group GSE + DOX: Grape seed extract with Doxorubicin, Group L-CA + DOX: L-Carnitine with Doxorubicin.

## Data Availability

The data presented in this study are available in the article.
